# Africa CDC: Establishing Integrated Surveillance and Laboratory Networks for Rapid Disease Detection and Response, Control, Prevention, and Clinical Care in Africa

**DOI:** 10.4102/ajlm.v6i1.638

**Published:** 2017-06-21

**Authors:** Timothy Amukele

**Affiliations:** 1 Department of Pathology, Johns Hopkins University School of Medicine, United States

## Introduction

There is little similarity between asbestos, HIV, and the severe acute respiratory syndrome coronavirus. However, their histories illustrate the three keys for effective detection and control of disease: clinical identification, laboratory detection, and public response. In diseases where all elements of this three-step framework were in place, the time from initial observation to appropriate public response tended to occur quickly. For example, in late November 2002, an atypical pneumonia that ultimately came to be known as severe acute respiratory syndrome made its appearance in China and a few other countries in East Asia. Once the World Health Organization was notified in February 2003^1^, a massive coordinated response was deployed. The causative agent was identified within two weeks^2^ and the disease was halted by effective quarantine in 42 days^3^.

If we want to support similar rapid responses to current and future epidemics, we need to create systems that foster the aforementioned three keys: clinical identification of disease, laboratory detection of disease, and public response to disease. The central piece of these three pillars is laboratory detection, which requires a good laboratory workforce and networks. In keeping with this central role, workforce and laboratory network development were the twin goals of the three-day Africa Centres for Disease Control (CDC) workshop held in Addis Ababa from 27 to 29 March 2017.

## Meeting Structure and Details

**FIGURE 1:**
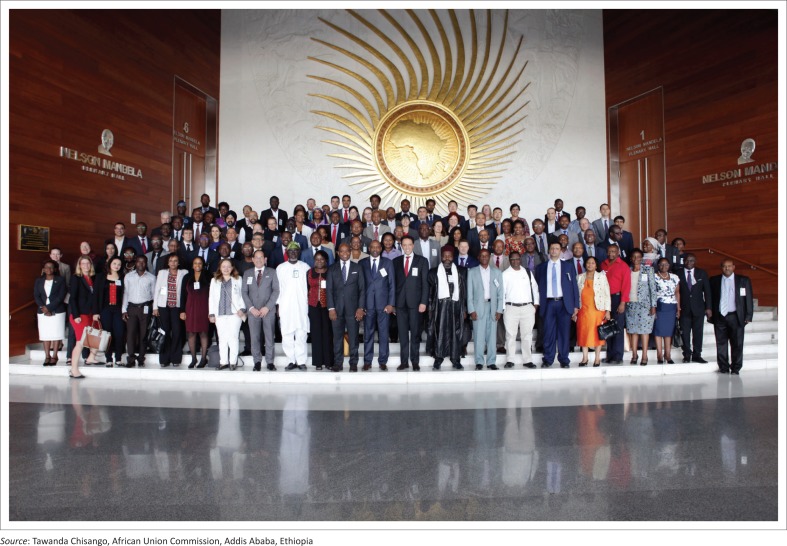
Attendees of the *‘Innovative Approaches to Establishing and Strengthening Regional Integrated Surveillance and Laboratory Networks for Disease Control, Prevention, and Clinical Care’* workshop. The workshop was jointly supported by the Africa CDC, the World Health Organization, and the African Society for Laboratory Medicine, and included participants from both the private and public sectors with a role in the laboratory sector in Africa.

The workshop, titled ‘*Innovative Approaches to Establishing and Strengthening Regional Integrated Surveillance and Laboratory Networks for Disease Control, Prevention, and Clinical Care*’, was jointly supported by the Africa CDC, the World Health Organization (WHO), and the African Society for Laboratory Medicine (Figure 1). The workshop had participants from both the private and public sectors who had a role in the laboratory sector in Africa. Each day was divided into brief morning talks focused on sharing best practices and case studies, and afternoon breakout sessions focused on addressing a list of key questions that were relevant to each day’s topic.

The first day of the meeting focused on laboratory networks currently in Africa. Two themes that were highlighted in the morning talks were the need for mapping of laboratories, as well as success stories in laboratory mapping from different African regions. Briefly, Dr Pascale Ondoa presented the LABNET scorecard^4^, which measures laboratory network preparedness, maturity, and adherence to regulations. Dr Brian Wood presented a report on a video-conferencing technology that trains primary care providers on how to treat complex diseases — the ‘Extension for Community Healthcare Outcomes (ECHO)’ model^5^. Dr Rebecca Martin shared the experience of establishing the East African Public Health Laboratory Network. Mr Adjane Kossivi Koura shared the impressive work of RESAOLAB, which has over 700 laboratories in its laboratory-strengthening network spread over seven west African countries^6^, and Dr Naima El Mdaghri talked about laboratory networks in North Africa.

The second day of the meeting focused on antimicrobial resistance (AMR). Four of six talks shared country examples of AMR best practices and the two others were by international partners. Dr Christopher Larson gave the first of the international-partner talks with an introduction of the WHO’s Global Antimicrobial Resistance Surveillance System (GLASS). GLASS, started in May 2015, is a harmonised system to collect worldwide AMR data. The second talk, given by Dr John Wilson, was an introduction to the Fleming Fund, a UK-funded initiative focused on helping low- and middle-income countries perform AMR surveillance as well as build capacity. The rest of the morning talks were examples of best practices from various African regions. Dr Tenaw Andualem discussed the AMR Control Strategy in Ethiopia, with emphasis on the key role that engaging the media plays in public education, Dr Kone Louis Penali shared the AMR Update and Control Strategy from Côte d’Ivoire, Dr Mohamed Genedy presented the AMR Update and Control Strategy in Egypt, and Dr Ondoa shared a talk from the CDC’s Senegal regional collaborating centre titled ‘*What Can a Framework for Surveillance Laboratory Networks for Antimicrobial Resistance in Five Regions of Africa Look Like? Role of Africa CDC Regional Collaborating Centres*’. The afternoon breakout sessions were also on the topic of AMR and were guided conversations focused on various aspects of establishing AMR surveillance, intervention and control, including the role of private and international partners. All groups returned with clear recommendations on the role Africa CDC could play in AMR going forward.

Day three of the symposium focused on two topics: laboratory workforce training and the role of the private sector (non-governmental organisations and private laboratories) in public health. As with the prior days of the workshops, the talks focused on best practices from various African regions. Dr Jane Carter discussed the critical role that non-governmental organisations play in advancing laboratory medicine and public health in Africa, through the framework of experiences from the African Medical and Research Foundation’s 50-year history. This was followed by Dr Tamrat Bekele, founder and CEO of International Clinical Laboratory (ICL), who discussed ICL’s support of the public healthcare sector in Ethiopia. ICL is a Joint Commission-accredited laboratory that has been in operation for 13 years in Addis Ababa. One example of many was the provision of full laboratory testing to Debrebirhan Hospital, located about 100 km east of Addis Ababa, as well as expanding microbiological culture to those public hospitals without the capacity to provide this service. Dr Trevor Peter next talked about the laboratory workforce development programmes. He introduced the audience to a new programme developed by the African Society for Laboratory Medicine for a community laboratory quality corps, which is focused on ensuring quality at the lower cadres of the health care system. Dr Samantha Dittrich shared the American Public Health Laboratory’s ‘Lab Manager Training’ programme, and Dr Patrick Nguku closed the presentations by presenting his work developing an epidemiology workforce through the Field Epidemiology and Laboratory Training Program in Nigeria^7^.

## Opportunities and Threats Highlighted in Interviews with Key Stakeholders

There was a palpable air of excitement and cautious optimism at this inaugural laboratory workshop of the Africa CDC. I interviewed two key stakeholders in an effort to capture some of their voices about the workshop and the way forward. The following were their responses to the question, ‘*What do you see as the way forward for strengthening laboratory networks and workforce development in Africa’*?

- Dr John Nkengasong (Inaugural Director, Africa CDC)
  ■ The first outcome of this meeting is the launch of the Africa CDC Regional Integrated Surveillance Laboratory Network (RISLNET). This will be a vehicle to drive networking in the five geographical regions. The five RISLNETs will be in Zambia, Kenya, Gabon, Nigeria, and Senegal. The goal is to have national public health institutes in each region anchored within the RISLNET. The close networking of the RISLNETs with national players will help to drive regionally appropriate plans for AMR, pandemic preparedness, and rapid response, among others.
  ■ The second outcome of this meeting is the establishment of the Africa CDC Anti-Microbial Resistance and Surveillance Network (Africa CDC AMRSNET). The goal would be to standardise the approach to AMR. The Africa CDC AMRSNET will work closely with WHO’s GLASS. Africa CDC AMRSNET will strive to achieve quality data across the region, establish mentorship programmes, and centres of excellence to drive AMR work.
  ■ The third outcome of this meeting is to highlight and improve the role of the private sector laboratories in supporting public health work in Africa. The private sector is well equipped to extend the reach and capacity of the public health role that Africa CDC is serving in the community. The most important thing for me is: ‘*is the African patient being served*?’
- Professor Alash’le Abimiku (Chair of the Board, African Society for Laboratory Medicine)
  ■ I am hoping that Africa CDC will use the wide array of best practices and resources that have been highlighted during this meeting to improve what it does as far as disease surveillance, prevention, control of outbreaks, laboratory workforce development, etc. Knowledge of the tools and possibilities out there will help Africa CDC to advocate more appropriately, based on a true understanding of the opportunities and challenges on the ground.
  ■ Secondly, I am hoping for better harmonisation of policies and programmes across borders. Even though this might require modification of what individuals and institutions are doing, this work is critical because we as Africans will have more political traction globally as a collective voice rather than singly. A good example of such collaboration is how we utilise the three to four biosafety level 4 laboratories in the continent. If we all work together within our respective regions we can take advantage of existing infrastructure rather than building from scratch. We all need the mindset that harmonisation, networking, and collaboration are value-added.
  ■ The Africa CDC has an incredible leader that is taking the advantage of this meeting to bring the best minds and ideas to the table. The success of the regional collaborating centres will depend largely on their ability to continue to think in this open-minded, proactive, and inclusive way.

## Summary and Next Steps

The goal of the Africa CDC is to, in their own words, ‘safeguard Africa’s health’. Achieving this goal on a population level requires three things: clinical acumen to diagnose disease, coordinated laboratory networks to diagnose disease, and an appropriate response to control disease. The workshop in March in Addis Ababa focused primarily on building and sustaining laboratory networks to aid disease detection and control. While there were many excellent ideas that came out of the breakout sessions suggesting various ways forward for the Africa CDC, what struck me was the abundance of positive examples from various regions of Africa. These examples from the private and public sector are already effective in the region, and have in some cases been sustained for decades. Success is inevitable if the Africa CDC serves as the custodian of these success models. The *African Journal of Laboratory Medicine* encourages submissions that highlight these examples of best practices.
